# Syncope in a Patient With an Extensive Thoracolumbosacral Myxopapillary Ependymoma

**DOI:** 10.7759/cureus.95457

**Published:** 2025-10-26

**Authors:** Alice S Wang, Nicole A Nguyen, Louis Reier, Jessica Dally, Carolyn S Leach, Ahmad Ibrahim, Dan E Miulli

**Affiliations:** 1 Neurological Surgery, Riverside University Health System Medical Center, Moreno Valley, USA; 2 Neurological Surgery, Arrowhead Regional Medical Center, Colton, USA; 3 Pathology, St. George's University School of Medicine, St. George’s, GRD; 4 Neurological Surgery, California University of Science and Medicine, Colton, USA; 5 Pathology, Arrowhead Regional Medical Center, Colton, USA; 6 Pathology and Laboratory Medicine, Arrowhead Regional Medical Center, Colton, USA

**Keywords:** adult hydrocephalus, elevated intracranial pressure, myxopapillary ependymomas, syncope workup, ventriculomegaly

## Abstract

Myxopapillary ependymomas are most commonly found involving the conus medullaris, cauda equina, and filum terminale, causing lower extremity symptoms, whereas atypical presentations such as syncope are rare. A 20-year-old woman presented with multiple syncopal episodes, headache, visual changes, and lower extremity paresis. Imaging revealed a T10-S2 myxopapillary ependymoma and mild ventriculomegaly. The patient underwent subtotal debulking, and histopathologic examination confirmed a WHO Grade II myxopapillary ependymoma. Postoperatively, her symptoms resolved. This case highlights the importance of evaluating spinal pathology in patients with atypical presentations of syncope.

## Introduction

Myxopapillary ependymomas (MPEs) are rare intradural, extramedullary spinal tumors, constituting approximately 13% of all spinal ependymomas. They predominantly occur in the conus medullaris and filum terminale regions [1]. The exact etiology of MPEs remains unclear. They are believed to originate from ependymal glial cells in the filum terminale, though the precise pathogenesis is still under investigation [2]. Clinically, MPEs present with symptoms resulting from the mass effect on adjacent structures, particularly the nerve roots of the cauda equina. Common manifestations include low back pain, radiculopathy, paresis, paresthesia, bowel and urinary incontinence, and gait instability [3]. Radiographically, MPEs typically show contrast enhancement on T1-weighted MRI and hyperintensity on T2-weighted images. Histologically, they may exhibit microcystic, solid, hemorrhagic, or hyaline components [4]. Despite their typically slow growth, MPEs are classified as WHO Grade II tumors in the 2021 WHO Classification of Tumors of the Central Nervous System [5]. Surgical resection is the primary treatment for MPEs. Achieving gross total resection is associated with a favorable prognosis, with five-year survival rates exceeding 98%. In contrast, subtotal resection carries a higher risk of local recurrence and progressive neurological deficits [5]. If only subtotal resection can be achieved, the prognosis is less favorable, with an increased risk of local recurrence and persistent or worsening neurological deficits. Adjunctive therapies may include chemotherapy or radiation therapy.

Atypical presentations of MPEs, though rare, have been documented. These include symptoms such as headache, blurry vision, diplopia, and neck stiffness. Diagnostic workup may reveal findings such as afferent pupillary defects, hemianopia, papilledema, elevated intracranial pressure, superficial siderosis, or subarachnoid hemorrhage [2,6-8]. Surgical resection of MPEs in the thoracolumbar spine often results in improvement or resolution of symptoms [2,6,9]. Intracranial findings may arise due to tumor cells, inflammatory cells, and elevated protein content in cerebrospinal fluid, which can interfere with normal cerebrospinal fluid absorption [9].

We present the case of a 20-year-old woman who experienced recurrent episodes of syncope, along with both typical and atypical features of MPE. Syncope associated with subtle ventriculomegaly should prompt spinal evaluation. Imaging revealed an extensive T10-S2 myxopapillary ependymoma, contributing to mild ventriculomegaly and suspected communicating hydrocephalus. The patient demonstrated clinical improvement following surgical debulking of the mass.

## Case presentation

A 20-year-old woman presented with multiple episodes of syncope, accompanied by a non-localizing headache characterized by intermittent dull pain without a specific location, and intermittent blurry vision with dark floaters, which she described as spots moving across her field of vision. She also reported an inability to ambulate due to bilateral lower extremity paresis and radiculopathy persisting for one month. Neurological examination revealed 4/5 motor strength on the Medical Research Council scale in both lower extremities, diminished sensation in the bilateral lower limbs, hyporeflexia, and an unsteady gait. Ophthalmologic evaluation demonstrated bilateral optic disc edema and papilledema.

**Figure 1:**
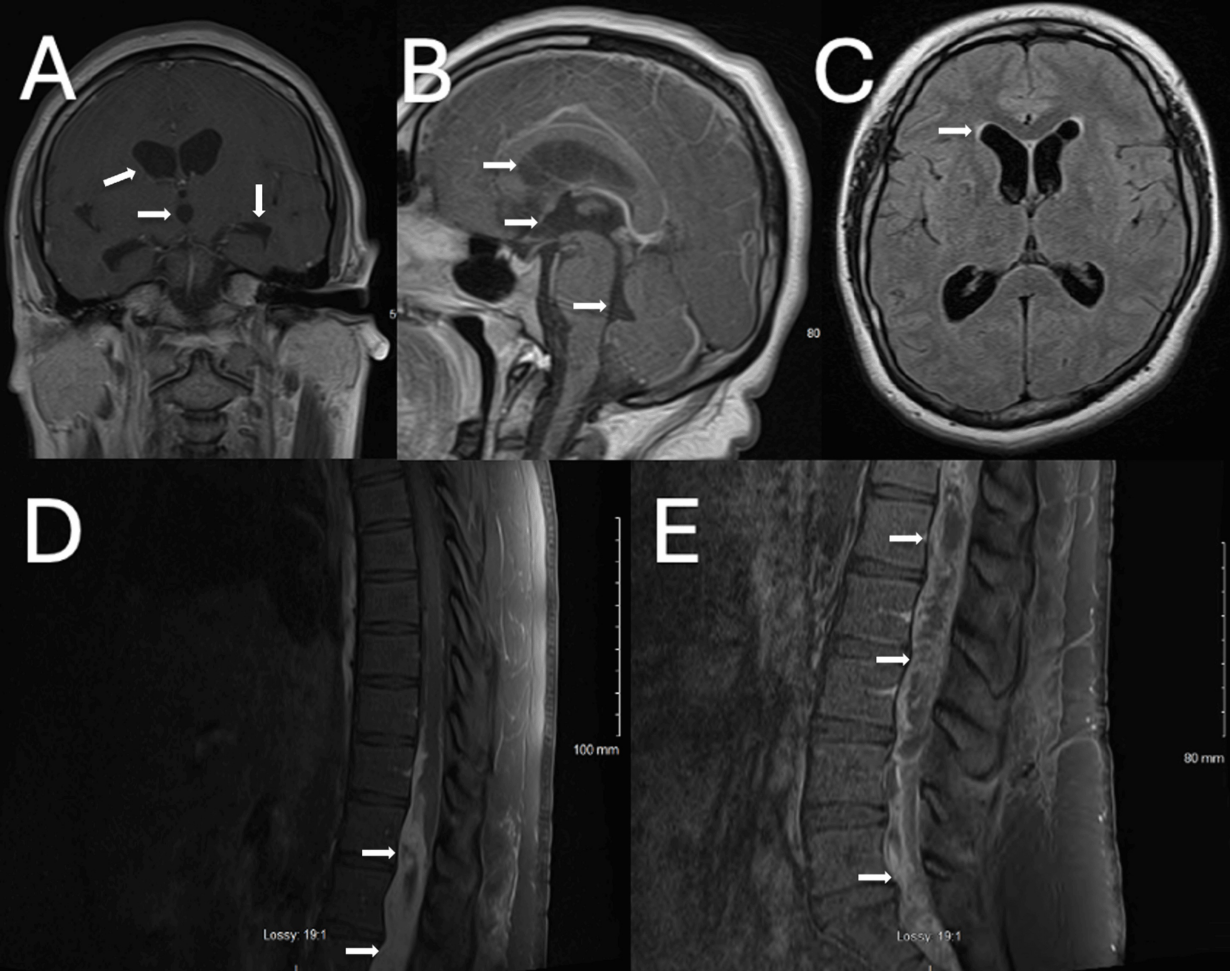
(A) Preoperative computed tomography (CT) of the head without contrast shows enlarged lateral ventricles, temporal horns, and third ventricle (white arrows). (B) Preoperative magnetic resonance imaging (MRI) of the brain without contrast again shows lateral ventricle, third ventricle, and fourth ventricle ventriculomegaly (white arrows) without any obstructive lesions, suggesting communicating hydrocephalus. (C) Preoperative MRI brain T2 FLAIR does not show transependymal flow (white arrow). (D-E) Preoperative MRI thoracic and lumbar spine T1 with contrast show an intradural intramedullary heterogeneously enhancing lesion spanning from T10 to S2 (white arrows) with compression on the spinal cord, conus medullaris, cauda equina, and filum terminale with invasion of neural foramina at multiple levels. FLAIR: fluid-attenuated inversion recovery.

Noncontrast computed tomography (CT) of the head revealed enlargement of the lateral ventricles, temporal horns, third ventricle, and fourth ventricle, without evidence of obstructive lesions, indicating communicating hydrocephalus. MRI of the brain using the FIESTA (Fast Imaging Employing Steady-State Acquisition) sequence showed no evidence of aqueductal stenosis or obstructive masses (Figure 1A-C). MRI of the thoracic and lumbar spine with contrast showed an intradural, intramedullary, heterogeneously enhancing lesion spanning from T10 to S2, with compression of the spinal cord, conus medullaris, cauda equina, and filum terminale, as well as invasion of neural foramina at multiple levels (Figure 1D-E).

**Figure 2:**
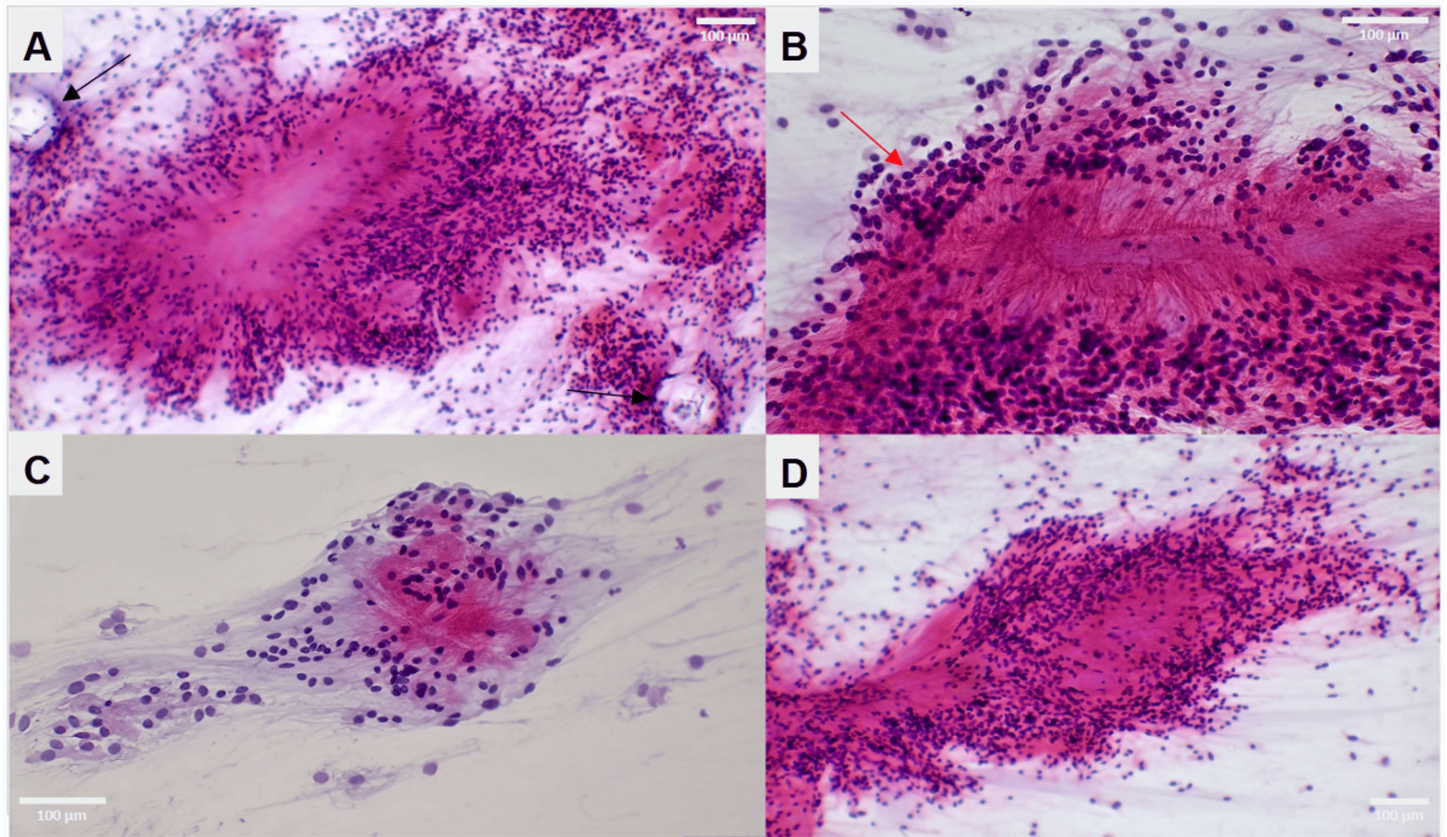
Squash prep of frozen sections, H&E. (A) 100x magnification. The tumor demonstrates a radial arrangement of cells around a hyalinized core with palisading nuclei. This is characteristic of myxopapillary ependymoma. Pseudorosette formations are present at the periphery (black arrows). (B) High-power view (200x magnification) showing thin fibrous strands radiating from a hyalinized core. Nuclei are located along the periphery of these strands, exhibiting open chromatin and prominent nucleoli (red arrow). (C) High-power view (200x magnification) highlighting nuclei with open (vesicular) chromatin and prominent nucleoli, set within a fibrous, myxoid stroma. Nuclei are arranged in a pseudorosette configuration within a delicate, whorled myxoid stroma. (D) 100x magnification. Nuclei are arranged along the periphery of fibrous strands in a radial configuration, producing an appearance reminiscent of a “cat’s tail.”

**Figure 3:**
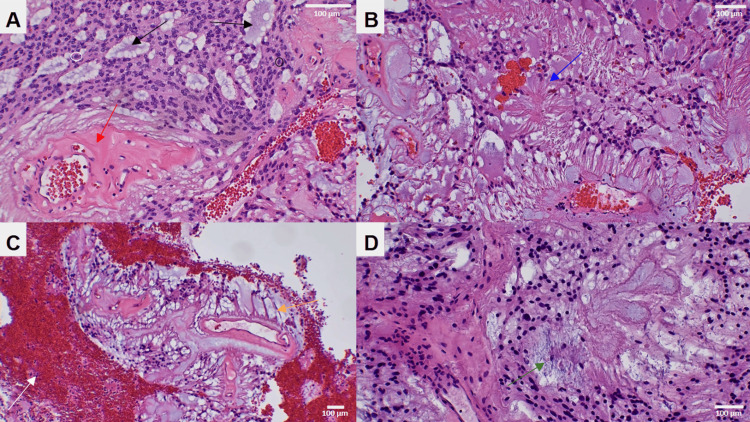
Tumor, H&E. (A) High-power view (200x magnification) demonstrating enlarged, vesicular nuclei with nuclear clefting (black circle) and occasional mitotic figures (white circle). Basophilic myxoid stroma is present within microcystic spaces (black arrows), along with notable perivascular hyalinization (red arrow). (B) High-power view (100x magnification) depicting the fibrous, palisading architecture of the tumor, arranged in a radial configuration (blue arrow). (C) Low-power view (40x magnification) illustrating a radial arrangement of elongated tumor cells around a hyalinized fibrovascular core in a palisading pattern (orange arrow). Areas of hyalinization, microcystic spaces, and prominent hemorrhage (white arrow) are also observed. (D) Frozen section, H&E. High-power view (100x magnification) demonstrating microcystic spaces and fibrous strands within the tumor (green arrow).

To prevent further neurological decline, surgical intervention was offered. However, due to the extent of tumor invasion, gross total resection was not achievable. Therefore, the patient underwent decompressive T10-L5 bilateral expansile laminoplasty via a posterior approach for debulking of the thoracolumbosacral mass. Intraoperatively, the mass was identified as soft, intradural, and intramedullary, with extensive adhesions to all intraspinal elements, including infiltration of the conus medullaris and neural foramina at multiple levels. There were no intraoperative changes in neuromonitoring (somatosensory evoked potentials, motor evoked potentials, and electromyography). Intraoperative pathology suggested a myxoid ependymoma. Final histopathologic examination confirmed a myxopapillary ependymoma (WHO Grade II). Immunohistochemical staining was positive for GFAP and S100 and negative for EMA and D2-40 (Figure 2A-D and Figure 3A-D). Cerebrospinal fluid analysis revealed a glucose concentration of 26 mg/dL (normal range: 40-70 mg/dL), a protein concentration of 2,314 mg/dL (normal range: 15-45 mg/dL), and negative cytology, indicating no leptomeningeal infiltration.

**Figure 4:**
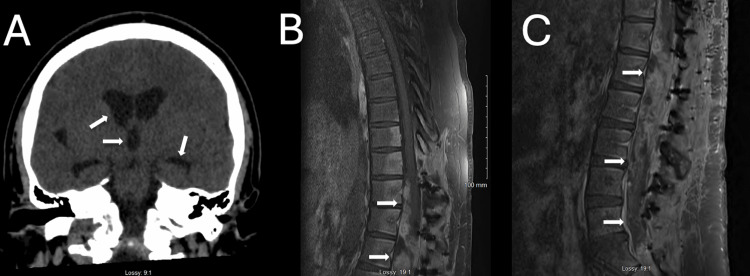
(A) Postoperative computed tomography head without contrast shows stable lateral ventricles, third ventricle, and temporal horns (white arrows). (B) Postoperative magnetic resonance imaging thoracic and lumbar spine with contrast show postoperative changes and residual tumor (white arrows).

Postoperative noncontrast CT of the head demonstrated stable third ventricle and temporal horn sizes. Postoperative contrast-enhanced MRI of the thoracic and lumbar spine revealed residual tumor at the surgical site, measuring approximately (size), which was consistent with postoperative changes (Figure 4A-C). The patient was discharged on postoperative day 16 with complete resolution of headache and visual disturbances, full recovery of motor strength, and improved sensation in both lower extremities. 

## Discussion

Myxopapillary ependymomas are rare spinal tumors most commonly arising in the conus medullaris, cauda equina, or filum terminale [1]. Typical symptoms result from mass effect on local nerve roots and include lower back pain, radiculopathy, motor weakness, sensory deficits, and bowel or bladder dysfunction [3]. Rarely, these tumors may cause atypical intracranial symptoms such as headache, visual changes, or syncope, likely due to impaired cerebrospinal fluid (CSF) absorption and communicating hydrocephalus [2,6,9]. In this patient, recurrent syncope was likely secondary to transient elevations in intracranial pressure from impaired CSF absorption, a phenomenon previously described in cases of spinal myxopapillary ependymoma [2,6,9].

Imaging findings may lag behind clinical improvement due to delayed normalization of CSF dynamics and brain compliance after tumor decompression. Gross total resection of extensive myxopapillary ependymomas is often limited by adherence to neural elements and invasion of the neural foramina. Subtotal resection followed by adjuvant radiotherapy to the tumor bed (typically 49-56 Gy) provides good local control and neurological outcomes [10]. The role of chemotherapy is limited and should be individualized, primarily for cases with leptomeningeal dissemination or recurrence [10].

To our knowledge, syncope as a presenting feature of extensive thoracolumbosacral myxopapillary ependymoma has been reported only rarely. This case highlights the importance of evaluating spinal pathology in atypical syncope and emphasizes the need for serial MRI follow-up after intervention to assess recurrence.

## Conclusions

This case underscores the importance of comprehensive evaluation in patients presenting with atypical neurological symptoms, including syncope, headache, and mild ventriculomegaly. Even subtle radiographic findings should prompt further spinal imaging when neurological deficits are present. Early diagnosis and timely surgical intervention, followed by appropriate adjuvant therapy, are crucial to prevent severe neurological morbidity and to optimize functional recovery. These patients require close surveillance and coordinated multidisciplinary care.
